# Internalizing Symptoms as Predictors of School Absenteeism Severity at Multiple Levels: Ensemble and Classification and Regression Tree Analysis

**DOI:** 10.3389/fpsyg.2019.03079

**Published:** 2020-01-21

**Authors:** Mirae J. Fornander, Christopher A. Kearney

**Affiliations:** Department of Psychology, University of Nevada, Las Vegas, Las Vegas, NV, United States

**Keywords:** absenteeism severity, truancy, ensemble analysis, classification and regression tree analysis, youth internalizing, risk variables

## Abstract

School attendance problems are highly prevalent worldwide, leading researchers to investigate many different risk factors for this population. Of considerable controversy is how internalizing behavior problems might help to distinguish different types of youth with school attendance problems. In addition, efforts are ongoing to identify the point at which children and adolescents move from appropriate school attendance to problematic school absenteeism. The present study utilized ensemble and classification and regression tree analysis to identify potential internalizing behavior risk factors among youth at different levels of school absenteeism severity (i.e., 1+%, 3+%, 5+%, 10+%). Higher levels of absenteeism were also examined on an exploratory basis. Participants included 160 youth aged 6–19 years (*M* = 13.7; SD = 2.9) and their families from an outpatient therapy clinic (39.4%) and community (60.6%) setting, the latter from a family court and truancy diversion program cohort. One particular item relating to lack of enjoyment was most predictive of absenteeism severity at different levels, though not among the highest levels. Other internalizing items were also predictive of various levels of absenteeism severity, but only in a negatively endorsed fashion. Internalizing symptoms of worry and fatigue tended to be endorsed higher across less severe and more severe absenteeism severity levels. A general expectation that predictors would tend to be more homogeneous at higher than lower levels of absenteeism severity was not generally supported. The results help confirm the difficulty of conceptualizing this population based on forms of behavior but may support the need for early warning sign screening for youth at risk for school attendance problems.

## Introduction

School attendance problems are a worldwide phenomenon linked to a plethora of academic, social, and physical and mental health problems in children and adolescents (Kearney et al., 2019a, b). Factors that elevate risk of school attendance problems are myriad as well and are often grouped into child-, parent-, family-, peer-, school-, and community-based variables (e.g., Havik et al., 2015). Child-based risk factors of school attendance problems include extensive work hours outside of school, grade retention, office disciplinary referrals, low school commitment and engagement, poor health or academic proficiency, problematic interpersonal relationships, substance use, and underdeveloped social and academic skills, among others (Kearney, 2008; Ekstrand, 2015; Gubbels et al., 2019). Other child-based risk factors of school attendance and academic achievement problems, as well as later school dropout, have involved various psychopathological conditions and symptoms (Macklem, 2014; Parr and Bonitz, 2015; Kearney, 2016).

School attendance problems have been linked historically to a variety of internalizing and externalizing behavior problems and disorders, most notably anxiety and mood disorders and disruptive behavior disorders (Kearney and Albano, 2004; Jones et al., 2019). Internalizing problems common to this population include general, social, and separation anxiety as well as worry, fear, depression, somatic complaints, fatigue, social withdrawal, sleep disturbance, and self-consciousness (Egger et al., 2003; Maynard et al., 2015; Gonzálvez et al., 2019). Externalizing problems common to this population include non-compliance, defiance, verbal and physical aggression, temper tantrums, refusal to move, running away from school or home, and antisocial and disruptive behavior at school and elsewhere (Ingul et al., 2012; Kearney, 2019). In addition, internalizing and externalizing problems are highly comorbid within and across each set in this population (Hankin et al., 2016; Finning et al., 2019).

In recent years, researchers have endeavored to move toward more detailed, nuanced, and sophisticated profiles of psychopathology in youth with school attendance problems, particularly with respect to internalizing behaviors and their treatment (Ek and Eriksson, 2013; Crawley et al., 2014; Fiorilli et al., 2017; Maynard et al., 2018). For example, researchers have found that depression and less prosocial behaviors are often primary features of anxious youth with school attendance problems (Pflug and Schneider, 2016; Sibeoni et al., 2018; Tekin et al., 2018). In addition, others have associated school attendance problems linked with internalizing behaviors to key profiles surrounding optimism/pessimism, positive/negative affect, social functioning, and anxiety severity (Gonzálvez et al., 2016, 2019; Fernández-Sogorb et al., 2018; Sanmartín et al., 2018).

Researchers have also endeavored to link specific psychopathological symptoms to various levels of school absenteeism severity. For example, Lawrence et al. (2019) found that students with a mental disorder displayed less school attendance than students without a mental disorder, missing 11.8 school days in years 1–6, 23.1 days in years 7–10, and 25.8 days in years 11–12. In addition, for those students with a mental disorder, absences due to a particular disorder accounted for 13.4% of all days absent from school (rising to 16.6% in years 11–12). Skedgell and Kearney (2016) also examined internalizing symptoms among youth with 0–14% and 15–100% absenteeism severity, finding the latter group (and particularly those at 20–39%) to display significantly more general and separation anxiety and depression. Stempel et al. (2017) similarly compared youth who had missed less than versus more than 15 days of school, finding that more chronic absenteeism was associated with more adverse childhood experiences such as financial hardship, divorce, parental incarceration, domestic or neighborhood violence, and family mental disorder or substance use.

A link between specific psychopathological symptoms and other risk factors with various levels of school absenteeism severity has important potential implications beyond basic research and classification. Certainly such a link can inform medical and mental health professionals who address youth with school attendance problems, and assessment and intervention protocols can be variously adapted to cases of mild/moderate versus chronic/severe absenteeism (Heyne et al., 2002; Kearney and Albano, 2018). Many school-based professionals and districts also distinguish between students with less severe and more severe academic and behavioral problems as they work to optimize limited intervention resources (McIntosh et al., 2010; August et al., 2018). Indeed, many schools have been forced to take on the role of mental health care and have thus sought out ways to screen for various mental health problems (Merikangas et al., 2011; Stiffler and Dever, 2015). Suggestions for what mental health symptoms relate to various levels of absenteeism severity would, for example, be helpful in this regard (Dowdy et al., 2015).

The need for more informed mental health screening in schools dovetails nicely with recent theoretical frameworks of school attendance problems that focus in part on multi-tiered interventions. Many school districts have adopted multi-tiered systems of support (MTSS) models for prevention and intervention of mental health concerns (Splett et al., 2018). MTSS models typically focus on prevention (Tier 1), early intervention for emerging, acute, or mild to moderate problems (Tier 2), and intensive intervention for chronic and severe problems (Tier 3) (Eagle et al., 2015). MTSS models can apply to a wide variety of academic, social, and behavioral problems, including those with internalizing behavior problems (Weist et al., 2018).

Kearney and Graczyk (2014) and Kearney (2016) were the first to apply MTSS principles to school attendance problems. In this model, Tier 1 strategies focus on enhancing functioning and schoolwide attendance and on preventing school attendance problems for all students, Tier 2 strategies focus on students with emerging, acute, or mild to moderate school attendance problems, often to reintegrate them to school, and Tier 3 strategies focus on students with chronic and severe school attendance problems, often to provide alternative pathways to graduation. Specific interventions may be matched to each tier based on absenteeism severity and degree of risk and contextual factors to help school personnel and others identify individualized responses (Freeman et al., 2016; Kearney, 2016; Elliott and Place, 2019).

As mentioned, MTSS models are increasingly adapted to a wide variety of academic, social, and behavioral problems, including now school attendance problems. A particular challenge for advocates of these models, however, has been to demarcate tiers within the system. A distinction between Tier 1 and Tier 2, for example, indicates a distinction between less problematic and more problematic behavior such as school absenteeism (Pullen and Kennedy, 2019). Unfortunately, no consensus distinction currently exists in this regard (Lyon and Cotler, 2007; Spruyt et al., 2016; Chu et al., 2018). In addition, distinctions between Tier 2 and Tier 3 remain variable. School attendance problems are sometimes considered to be chronic and severe (Tier 3) at a 10% threshold (DePaoli et al., 2015). Skedgell and Kearney (2016, 2018) found that risk factors for higher severity levels of absenteeism tended to be more homogeneous than risk factors at lower levels of absenteeism. However, data to support a Tier 2-Tier 3 distinction remain needed (Conry and Richards, 2018).

The present study aimed to identify potential internalizing symptom risk factors among youth at different levels of school absenteeism severity (i.e., 1+%, 3+%, 5+%, 10+%). Such differentiations might help inform distinctions between tiers in an MTSS model of school absenteeism. In accordance with recent calls to employ machine learning-based methods to examine risk factors for school absenteeism (Chung and Lee, 2019; Sansone, 2019), two sets of statistical approaches were utilized. Ensemble analysis, including chi-square adjusted interaction detection (CHAID), support vector machines, and neural network analyses, is a non-parametric method that combines multiple algorithmic models or classifiers to produce a single best model for a given data set (Berk, 2006). In addition, classification and regression tree (CART) analysis is a non-parametric method that identifies comprehensive subgroups based on interactions among multiple risk factors or predictor variables (Lemon et al., 2003). These analyses are aimed to generate and not test hypotheses (Markham et al., 2013). Various levels of school absenteeism were examined, with a general expectation that risk factors at higher levels of absenteeism would be more homogeneous than risk factors at lower levels of absenteeism.

## Materials and Methods

### Participants

Participants included 160 youth aged 6–19 years (*M* = 13.7; SD = 2.9) and their families from an outpatient therapy clinic (39.4%) and community (60.6%) setting in southern Nevada, the latter from a family court and truancy diversion program cohort. The clinic cohort involved students referred to therapy services for absenteeism; the community cohort involved students given a truancy citation by school police for absenteeism and referred to an 8-week diversion program. Participants were primarily male (51.2%) and diverse with respect to ethnicity: Hispanic (51.0%), European-American (26.1%), Asian (8.9%), African American (6.4%), multiracial or biracial (4.5%), and other (2.5%). Most parents were married (44.6%); others were divorced (22.3%), separated (18.5%), never married (12.7%), or had another status (1.9%). Most fathers (48.0%) and mothers (59.9%) graduated high school. Participants missed a mean of 19.0% days of school (SD = 16.9) at time of assessment. Some youths were referred for treatment for school refusal behaviors (e.g., distress at school, morning misbehaviors designed to miss school, skipped classes, and tardiness) that did not include formal absences.

### Measures

The Revised Children’s Anxiety and Depression Scale (RCADS; Chorpita et al., 2000) is a 47-item self-report or parent-report measure of child internalizing behavior disorders with the following subscales and number of items: separation anxiety (7), social phobia (9), generalized anxiety (6), obsessive-compulsive (6), panic disorder (9), and major depression (10). Items are scored on a Likert-type 0–3 scale of agreement (never = 0, sometimes = 1, often = 2, always = 3). Internal consistency is good for each subscale, with Cronbach’s alpha between 0.78–0.88 (Chorpita et al., 2005). Cronbach’s alpha for RCADS items in the present study was 0.86. Confirmatory factor analysis indicated the 6-factor model is an adequate fit, with loadings from 0.51–0.79 (Chorpita et al., 2005).

School staff or parents provided absenteeism severity data in the form of number of full school days missed. Percentage of full school days missed was calculated by dividing the student’s total number of full school days missed by the number of days of school in that academic year, at the time of assessment, and then multiplying that number by 100. Assessments were conducted at different points throughout the academic year.

### Procedure and Data Analyses

Participants were recruited from a specialized outpatient therapy clinic or community setting. Participants in the community setting were referred to family court or a truancy diversion program by their school or parent(s)/guardian(s) based on prior school absences. Following parent consent and child assent, measures that included the RCADS were administered to youth and their parent(s)/guardian(s) independently and in the presence of a research assistant. Spanish versions of the measures were available.

Ensemble analysis was utilized to identify potential family environment risk factors among youth with school attendance problems across different levels of school absenteeism. Ensemble analysis is the combination of multiple algorithmic models or classifiers to produce one, best model that can be applied to the data (Berk, 2006). These models have been shown to outperform standard parametric methods, primarily due to the automation of identifying interactions and non-linearities and the reduction of overestimations of a model’s predictive ability (Rosellini et al., 2018). Ensemble analysis can include many different statistical methods; the present study utilized CHAID decision trees, support vector machines, and neural network analyses. Predictors were examined collectively and independently. A multiple imputation method was utilized; different plausible imputed data sets were examined and combined results were obtained and reported here. Confusion matrices supported the use of CHAID decision trees. In addition, CART analyses were utilized to more specifically examine clusters of RCADS items associated with enhanced risk for a particular level of absenteeism severity (i.e., 1+%, 3+%, 5+%, 10+%). Other absenteeism levels were examined on an exploratory basis (i.e., 15+%, 20+%, 30+%, 40+%), as was latent class analysis for 0–10% and 10+% absenteeism. For brevity, significant results are reported. No gender differences were found with respect to RCADS Anxiety and Depression T-scores.

## Results

### Absenteeism: 1+%

For the CHAID analysis, the final collective tree-model that best differentiated youth with 1+% absenteeism from youth with <1% absenteeism correctly identified 99.6% of participants and identified one main risk factor: item 6 (nothing fun anymore; DEP). Item 6 scores of >0.0 indicated higher risk of 1+% absenteeism (69.3%); item 6 scores of 0.0 indicated lower risk (30.7%). The tree-model demonstrated higher sensitivity than specificity. Independent analysis revealed no significant predictors. CART item analysis similarly identified one subgroup at highest risk for 1+% absenteeism (node at 100.0%): endorsement of sometimes, often, or always on item 6 and endorsement of never on item 46 (scared if away from home overnight; SEP). The overall tree-model’s accuracy in predicting 1+% absenteeism was approximately 95.7%.

### Absenteeism: 3+%

For the CHAID analysis, the final collective tree-model that best differentiated youth with 3+% absenteeism from youth with <3% absenteeism correctly identified 83.7% of participants and identified one main risk factor: item 6 (nothing fun anymore; DEP). Item 6 scores of >0.0 indicated higher risk of 3+% absenteeism (53.4%); item 6 scores of 0.0 indicated lower risk (46.6%). The tree-model demonstrated higher sensitivity than specificity. Independent analysis of the predictors revealed that item 6 (*p* < 0.01, *F* = 12.19) and item 35 scores (*p* < 0.01, *F* = 7.81) significantly predicted 3+% absenteeism. With respect to item 35 (worry about what will happen; GAD), scores of 0.0 indicated higher risk (59.0%); scores of >0.0 indicated lower risk (41.0%). CART item analysis identified one main subgroup at highest risk for 3+% absenteeism (node at 100.0%): endorsement of sometimes, often, or always on items 6 (nothing fun anymore; DEP) and 38 (afraid to talk in front of class; SOP) as well as endorsement of never or sometimes on item 46 (scared if away from home overnight; SEP). The overall tree-model’s accuracy in predicting 3+% absenteeism was approximately 92.1%.

### Absenteeism: 5+%

For the CHAID analysis, the final collective tree-model that best differentiated youth with 5+% absenteeism from youth with <5% absenteeism correctly identified 76.7% of participants and identified one main risk factor: item 6 (nothing fun anymore; DEP). Item 6 scores of >0.0 indicated higher risk of 5+% absenteeism (53.4%); item 6 scores of 0.0 indicated lower risk (46.6%). The tree-model demonstrated higher sensitivity than specificity. Independent analysis of the predictors revealed that item 6 (*p* < 0.01, *F* = 12.19), 35 (*p* < 0.05, *F* = 6.30) and 38 scores (*p* < 0.05, *F* = 6.81) significantly predicted 5+% absenteeism. With respect to item 35 (worry about what will happen; GAD), scores of 0.0 indicated higher risk (59.0%); scores of >0.0 indicated lower risk (41.0%). With respect to item 38 (afraid to talk in front of class; SOP), scores of 0.0 indicated higher risk (61.3%); scores of >0.0 indicated lower risk (38.7%).

Classification and regression tree item analysis identified one main subgroup at highest risk for 5+% absenteeism (node at 100.0%): endorsement of never on item 17 (scared to sleep on own; SEP) and often or always on item 24 (with a problem, heart beats fast; PAN). The overall tree-model’s accuracy in predicting 5+% absenteeism was approximately 84.9%. Latent class analysis of <10% absenteeism revealed a primary cluster that contained 41% of cases. In this cluster, RCADS items 1–4, 7, 12, 13, 21, 25, and 30 (3 DEP, 2 GAD, 2 SOP, 1 PAN) were primarily endorsed as sometimes; all other items in this cluster were endorsed as never.

### Absenteeism: 10+%

For the CHAID analysis, the final collective tree-model that best differentiated youth with 10+% absenteeism from youth with <10% absenteeism correctly identified 58.5% of participants and identified one main risk factor: item 6 (nothing fun anymore; DEP). Item 6 scores of >0.0 indicated higher risk of 1+% absenteeism (52.3%); item 6 scores of 0.0 indicated lower risk (47.7%). The tree-model demonstrated higher sensitivity than specificity. Independent analysis of the predictors revealed that obsession/compulsions T-scores significantly predicted 10% of days missed (*p* < 0.01, *F* = 12.38). Obsession/compulsions T-scores of ≤48.0 indicated higher risk of 10+% absenteeism (57.8%); obsession/compulsions T-scores of >48.0 indicated lower risk (42.2%). In addition, endorsement of never on several items was also predictive of 10+% absenteeism: items 8 (worried when someone angry at me; SOP; 65.3%/34.7%), 9 (worry about being away from parents; SEP; 68.4%/31.6%), 29 (feel worthless; DEP; 66.7%/33.3%), 30 (worry about making mistakes; SOP; 67.6%/32.4%), 42 (have to do things over and over; OCD; 61.5%/38.5%), and 44 (have to do things in just the right way; 54.9%/46.1%).

Classification and regression tree item analysis identified one main subgroup at highest risk for 10+% absenteeism (node at 85.6%): endorsement of never on item 17 (scared to sleep on own; SEP). The overall tree-model’s accuracy in predicting 10+% absenteeism was approximately 84.2%. Latent class analysis of 10+% absenteeism revealed a primary cluster that contained 34% of cases. In this cluster, RCADS items 1, 4, 8, 21, and 30 (3 SOP, 1 DEP, 1 GAD) were primarily endorsed as sometimes; all other items in this cluster were endorsed as never.

### Absenteeism: Higher Levels

Chi-square adjusted interaction detection analyses were also conducted on an exploratory basis for absenteeism levels of 15+%, 20+%, 30+%, and 40+%. The final collective tree-model that best differentiated youth with 15+% absenteeism from youth with <15% absenteeism correctly identified 52.9% of participants and identified one main risk factor: item 6 (nothing fun anymore; DEP). Item 6 scores of >0.0 indicated higher risk of 15+% absenteeism (52.3%); item 6 scores of 0.0 indicated lower risk (47.7%). The tree-model demonstrated higher specificity than sensitivity. Independent analysis revealed no subscale scores to be significant predictors of 15+% absenteeism. In addition, endorsement of never on several items was also predictive of 15+% absenteeism: items 1 (worry about things; GAD; 60.9%/39.1%), 8 (worried when someone angry at me; SOP; 65.3%/34.7%), 9 (worry about being away from parents; SEP; 68.4%/31.5%), 25 (cannot think clearly; DEP; 66.9%/33.1%), and 29 (feel worthless; DEP; 66.7%/33.3%).

The final collective tree-model that best differentiated youth with 20+% absenteeism from youth with <20% absenteeism correctly identified 61.4% of participants and identified one main risk factor: item 6 (nothing fun anymore; DEP). Item 6 scores of >0.0 indicated higher risk of 1+% absenteeism (52.3%); item 6 scores of 0.0 indicated lower risk (47.7%). The tree-model demonstrated higher specificity than sensitivity. Independent analysis of the predictors revealed that item 42 significantly predicted 20+% absenteeism (*p* < 0.05, *F* = 6.58). Item 42 (have to do things over and over; OCD) scores of 0.0 indicated higher risk for 20+% absenteeism (61.5%); item 42 scores of >0.0 indicated lower risk (38.5%).

The final collective tree-model that best differentiated youth with 30+% absenteeism from youth with <30% absenteeism correctly identified 75.3% of participants and identified two main risk factors: item 8 (worried when someone angry at me; SOP) and separation anxiety subscale scores. Item 8 scores of >0.0 indicated higher risk of 30+% absenteeism (64.9%); item 8 scores of 0.0 indicated lower risk (35.1%). Separation anxiety T-scores of ≤61.0 indicated higher risk of 30+% absenteeism (53.1%); separation anxiety T-scores of >61.0 indicated lower risk (46.9%). The tree-model demonstrated higher specificity than sensitivity.

The final collective tree-model that best differentiated youth with 40+% absenteeism from youth with <40% absenteeism correctly identified 83.9% of participants and identified one main risk factor: item 28 (with a problem, feel shaky; PAN). Item 28 scores of 0.0 indicated higher risk of 40+% absenteeism (50.6%); item 28 scores of >0.0 indicated lower risk (49.4%). The tree-model demonstrated higher specificity than sensitivity.

## Discussion

The present study examined internalizing behaviors as potential predictors of various absenteeism severity levels. The findings revealed that one particular depression item (nothing much fun anymore) helped most to demarcate different severity levels, up to a point. In addition, a number of other internalizing items were predictive of various levels of absenteeism severity, but only in a negatively endorsed fashion. Overall, internalizing items that tended to be endorsed higher across less severe and more severe absenteeism severity levels included those relating to worry and fatigue. A general expectation that predictors would tend to be more homogeneous at higher than lower levels of absenteeism severity was not generally supported.

One particular item was found to consistently distinguish lower and higher levels of absenteeism severity at different benchmarks: item 6 (nothing is much fun anymore), which is an item on the RCADS depression subscale. Two general possibilities may exist for this finding. First, school attendance problems are indeed commonly associated with symptoms of depression, one of the rare consistent findings over several decades with respect to internalizing psychopathology in this population (Kearney, 1993; Egger et al., 2003; Gallé-Tessonneau et al., 2019). Depression is also commonly associated or comorbid with anxiety disorders in this population, making attempts at diagnostic classification difficult (Jones and Suveg, 2015). Antidepressant medication is recommended for many adolescents with school attendance problems, and cognitive-behavioral therapies for this population often focus on depression symptoms (Maynard et al., 2015; Londono Tobon et al., 2018; Melvin and Gordon, 2019).

Finning et al. (2019), in their meta-analysis of depression and school attendance problems, concluded that symptoms of depression are indeed common to many different types of school attendance problems. The authors also postulated several possible mechanisms for this association, such as social withdrawal, sleep disturbance, and low energy. Youth with school refusal behavior do tend to have social functioning problems and withdraw from friends and other peers at school (Havik et al., 2015; Gonzálvez et al., 2019). Others indeed show difficulties with sleep (including going to bed very late), energy, and physical activity (Ek and Eriksson, 2013; Hochadel et al., 2014; Mannino et al., 2019). However, each set of behaviors – social and sleep problems and school attendance problems – may precede the other in different cases (Kearney, 2019).

Second, the depression item noted above may also indicate a relative amount of boredom, frustration, burnout, or lack of self-efficacy with respect to the school environment or academic performance (Fiorilli et al., 2017). Finning et al. (2019) noted that another mechanism explaining depression and school attendance problems might be loss of motivation. Surveys of youth with school attendance problems or who have dropped out of school regularly reveal boredom with classes and the school environment as a key reason for leaving (Strand, 2014; Attwood and Croll, 2015; Kearney, 2016). Others have noted as well that youth with learning disorders can become frustrated and eventually miss school (Redmond and Hosp, 2008). Poor school climate or school-based curricula perceived as tedious or inflexible by students are associated with school attendance problems as well (Hendron and Kearney, 2016; Maxwell, 2016; Wang and Degol, 2016). Interestingly, the finding regarding item 6 disappeared at particularly high levels of absenteeism severity (i.e., 30+% and 40+%), possibly suggesting that some youth discovered outside-of-school avenues to boost enjoyment (Kearney and Albano, 2018).

A key finding of the present study was that lack of endorsement of several anxiety items was what most predicted higher absenteeism severity levels. The findings also indicated substantial variability with respect to individual items. One possibility is that higher absenteeism severity levels are associated more with externalizing than internalizing symptoms (Maynard et al., 2012). In addition, youth in the present study were examined at different points of the academic year, but anxiety levels may be more pronounced at the beginning of a year (Ingul and Nordahl, 2013). Higher levels of absenteeism severity also mean more time out of school and thus relief from school-based anxiety symptoms (Skedgell and Kearney, 2018). Other variables such as family or school environment may thus be better predictors of absenteeism severity (Fornander and Kearney, 2019).

The lack of endorsement and variability shown in the present study may also help confirm that reliance on various forms of specific behavior to identify classes of school attendance problems is quite difficult (Inglés et al., 2015). Kearney (2002) advocated for the term negative affectivity rather than specific symptoms of anxiety or depression among youth with school attendance problems to account for the vagaries of internalizing symptoms characteristic of this population. Indeed, historically, many researchers have focused on broad descriptors of emotional distress (e.g., dread, upset, misery) to describe youth who are reluctant to attend school (Kearney, 2001). Perhaps not surprisingly, the items that tended to be elevated more in the current study were those related to broader concepts such as worry and fatigue. Others have found considerable heterogeneity within and across classes of behavior among children with school attendance problems, and Kearney (2007) found that functions of school refusal behavior were superior to forms of behavior in predicting absenteeism severity.

Limitations of the present study should be noted. First, the sample was an eclectic one that ranged from having no formal school absences to having many school absences. Second, sample size constraints did not permit more nuanced analyses of absenteeism type, setting, or demographic or developmental differences, though studies generally indicate emotional distress across many absence types in this population (Finning et al., 2019). Third, the primary dependent measure was based on self-report, though these kinds of measures are commonly used for youth with internalizing symptoms (Chorpita et al., 2000). In related fashion, broader measures such as diagnostic interviews, behavioral observations, and parent and teacher reports were not used and may have provided more sophisticated information about participants’ internalizing symptoms.

## Conclusion

Despite these limitations, the present study may have some applicability to MTSS models of school absenteeism and how tiers within these models may be demarcated. Psychosocial screenings for anxiety and depression at early warning sign stages for problematic absenteeism may be advisable, and may help distinguish Tier 1 school attendance from emerging Tier 2 school attendance problems (Ingul et al., 2019). Findings from the present study may further support the need for preventative practices in this population as well, particularly for targeted practices aimed toward those with depressive symptoms (Werner-Seidler et al., 2017).

## Data Availability Statement

The datasets generated for this study are available on request to the corresponding author.

## Ethics Statement

The studies involving human participants were reviewed and approved by the Ethics Committee of the University of Nevada, Las Vegas. Written informed consent to participate in this study was provided by the participants’ legal guardian/next of kin.

## Author Contributions

Both authors revised and approved the submitted version. MF helped to collect the data, performed the initial analyses, and assisted in writing the manuscript. CK helped with data analysis, assisted in writing the manuscript, and supervised the study.

## Conflict of Interest

The authors declare that the research was conducted in the absence of any commercial or financial relationships that could be construed as a potential conflict of interest.
